# The Differential Diagnosis of Præfrontal and Cerebellar Tumours

**Published:** 1901-02-23

**Authors:** Arthur Maude


### HOSPITAL CLINICS AND MEDICAL PROGRESS.

### THE DIFFERENTIAL DIAGNOSIS OF PRÆFRONTAL AND CEREBELLAR TUMOURS.

By ARTHUR MAUDE.

The psychological changes occurring in cerebellar tumour are slight and late in manifestation (Williamson); on the other hand, in 50 cases analysed by him the mental symptoms in præfrontal lesions are usually well marked, and are frequently the earliest and most prominent signs.

The general mental condition in the subjects of intracranial tumour is an increasing drowsy torpitude in the day, frequently alternating with nocturnal excitability and restlessness. Lloyd draws attention to the slow reaction time of cerebration as significant of anterior frontal lesion: this condition is evidenced by slowness in response, though the answers to questions are correct and sensible: it is doubtful if this slow reaction is not presented at some period by all victims of intercranial tumour wherever situated. Jastrowitz again has claimed as a pathognomonic peculiarity of the præfrontal tumour a peculiar hilarity, or chuckling cheerfulness, hand in hand with increasing mental impairment; Oppenheimer and Brun corroborate this observation.

This condition is well marked in our example case: she is always smiling, and says she feels so happy. She reminds us of a person recovering from nitrous oxide. Her mental reaction is also extremely slow, and she has the great tendency to fall asleep characteristic of all cerebral tumours.

On the whole the English authorities, such as Michell Clarke and Williamson, support this view of Jastrowitz, Oppenheimer, Brun, and many others, that marked and early mental changes accompany lesion of the præfrontal area. The error in estimating all symptoms of doubtful intracranial tumours is to regard any single symptom as pathognomonic or of absolute value. The difficulty "lies in the common occurrence of mental changes in tumours of other regions of the brain, so that in any individual case it is hard to say whether the mental symptoms present are more than may accompany an intracranial tumour in other situations" (Michell Clarke).

The patellar reflexes were, in the case of Nellie I., completely absent from the first. The evidence is of no great positive value. In affection of the motor area the knee-jerks are seldom lowered; the reflex becomes increased on the opposite side to that of the tumour, and remains unchanged on the other. In cerebellar tumour it is frequently abolished, and in præfrontal tumour, though sometimes abolished, it is more often normal or increased.

"The state of the knee-jerks in tumour of the posterior cranial fossa is an uncertain quantity" (Aldren Turner, Brain," 1898, p. 342).

The gait of our patient could never be described as ataxic, reeling, or tending to circular movement towards either side. It has been simply feeble and shuffling, becoming gradually more feeble. Ataxic gait, or gait with broad base, is naturally significant of cerebellar disease, but it has been found in affection of the præfrontal region, according to Bruns; and according to Williamson in a high proportion of cases, 14 out of 50.

There is no noticeable difference between the power of the legs; the grasp and use of the upper extremities are quite unchanged in our patient.

The only suggestion of paralysis which has so far occurred in N. I. is an incomplete ptosis of the right eyelid, which was transient. Admitting this sign to evidence it hints at some focal lesion in the interpeduncular space, which has begun to affect the third nerve in part: such a position of tumour is likely to be associated with a grave and early form of optic neuritis. The external muscles of the eye and the iris show no paresis.

The greatest difficulty always lies in the differential diagnosis of tumours of the frontal and cerebellar regions, and in the localisation of the rarer sites, the optic thalamus, the corpus callosum, and the lateral ventricles. Extreme pressure from growth or fluid in the lateral ventricles is prone to produce a very profound general paresis, so that cases have been mistaken for general paralysis of the insane.

In tumours of the corpus callosum the headache is vertical, while the mental symptoms, which occur early, are usually followed by double hemiplegia at no late date. At some period in the history of præfrontal tumours general or localised convulsions are frequently noted: they are very rare in tumours of the cerebellum.

In placing the locality of a cerebral tumour we have far greater difficulty than we have presented in a defined stationary lesion like an intracranial hæmorrhage, a lesion which if not defined and stationary speedily causes death. The variations in pathological structure, in rate of growth, in amount of surrounding infiltration, in the production of secondary pressure on venous outlets, all tend to mask the effects produced locally on the areas and connecting paths of the brain. A slow-growing tumour may, whatever its position (unless that position involves absolutely vital centres) produce few symptoms, because the surrounding parts have time to adjust themselves to their new pressures and their new surroundings.

It would therefore be of primary importance, along with the initial position of a growth, to determine its pathological character. We are, unfortunately, but seldom able to do so. In rare cases we may be fairly sure of secondary deposits of tubercle, sarcoma, or carcinomata. Even syphilitic deposits frequently play us false, because syphilis of the brain is of late syphilitic history, is often heralded by an imperfect and even absent initial history, and because that standby of diagnosis, the behaviour of the disease under antisyphilitic remedies, is not to be trusted. Improvement of a case of tumour under the administration of iodide is no proof of its syphilitic nature. Sequin has shown the value of the drug in the temporary improvement of gliomata of the cerebellum, and Allen Starr in those of the cerebrum.

It is of peculiar importance to differentiate between præfrontal and cerebellar tumour, since now the question of operation on all cases of intracranial growths should be at least considered. Among the many discordant notes struck in the discussion at the British Medical Association meeting of 1898 there was comparative unanimity in the belief that (excluding gummata) hardly any intracranial tumours ever disappear or "heal," even when tubercular.

Since we know that almost every case of new growth in the brain is progressive, and ultimately fatal, and since the risks of exploratory opening of the skull are reduced as much as they now are in the hands of experienced surgeons, every case of certain tumour, excluding some tumours of the base, should be submitted to exploration. If the growth is not to be found, or is found to be infiltrating in character or otherwise irremovable, the mere opening of the skull may improve the wretched condition of the patient, and in particular may arrest the optic neuritis. But the difficulties and dangers of operation on cerebellar tumours are far greater than those of operation on the anterior lobe. And we have to add to this risk the difficulty adduced by Dr. Byron Bramwell, that in cases of apparent tumour of cerebellum there is a certain proportion which are in reality those of localised meningitis, blocking the foramen of Majendie, and thus producing a dilated condition of the ventricles.

